# Diagnostic and Prognostic Biomarkers for Sepsis‐Associated Liver Injury: Current Status and Future Perspectives

**DOI:** 10.1155/grp/8707150

**Published:** 2026-03-16

**Authors:** Yao-zheng Cai, Jiang-tao Chen, Le-xin Fang, Yue-ping Ding

**Affiliations:** ^1^ Department of Intensive Care Unit, The Second Affiliated Hospital of Zhejiang Chinese Medical University, Hangzhou, Zhejiang, China, z2hospital.com

## Abstract

Sepsis‐associated liver injury (SALI) is a complication of sepsis that carries a notably poor prognosis in the intensive care unit (ICU). So far, there remains no specific consensus regarding the diagnostic criteria for SALI. The quest for biomarkers associated with SALI continues, as they are essential for both early diagnosis and prognostic assessment. Traditionally utilized biomarkers include alanine aminotransferase (ALT), aspartate aminotransferase (AST), and bilirubin; however, their lack of specificity and sensitivity has hindered the accurate diagnosis of SALI and the prediction of subsequent disease progression. Recently, novel biomarkers such as microRNAs, differentially expressed genes (DEGs), and high mobility group protein B1 (HMGB1) have been explored to enhance the early diagnosis and prognostic prediction of SALI. However, each of these biomarkers presents certain limitations. This review is aimed at summarizing the aforementioned biomarkers with the hope that future researchers will identify the most effective markers for diagnosing SALI.

## 1. Introduction

Sepsis remains a leading cause of mortality in intensive care units. In China, approximately 25% of ICU patients are affected by sepsis, which significantly worsens prognosis [1]. The liver plays a pivotal role in the pathophysiology of sepsis: It serves as a critical barrier against microbial invasion while simultaneously acting as a primary target of systemic inflammation due to its central role in clearing pathogens, toxins, and inflammatory mediators [2]. Liver dysfunction in sepsis is an independent predictor of poor outcome; once overt liver injury or failure occurs, mortality rates range from 54% to 68% [3].

The pathogenesis of sepsis‐associated liver injury (SALI) is complex and involves multiple overlapping mechanisms, including direct endotoxin‐induced hepatocyte damage, mitochondrial dysfunction, excessive reactive oxygen species production, lipid peroxidation, massive release of damage‐associated molecular patterns (DAMPs) (e.g., HMGB1), and activation of inflammatory cascades such as NF‐*κ*B, impaired peroxisome proliferator‐activated receptor‐*α* (PPAR‐*α*) signaling, and NLRP3 inflammasome–mediated pyroptosis. A comprehensive understanding of these pathways is essential for identifying reliable diagnostic and prognostic biomarkers.

Although no internationally unified diagnostic criteria for SALI currently exist, three definitions are most widely accepted and consistently applied in contemporary clinical research and multicenter studies. The first, and most globally adopted, is a *l* *i* *v* *e* *r* *S* *e* *q* *u* *e* *n* *t* *i* *a* *l* *O* *r* *g* *a* *n* *F* *a* *i* *l* *u* *r* *e* *A* *s* *s* *e* *s* *s* *m* *e* *n* *t* (*S* *O* *F* *A*) *s* *c* *o* *r* *e* ≥ 2, defined exclusively by serum total *b* *i* *l* *i* *r* *u* *b* *i* *n* > 34 * μ*mol/L (2.0 mg/dL); this remains the standard in the Sepsis‐3 framework and Surviving Sepsis Campaign guidelines. The second requires the combination of *t* *o* *t* *a* *l* *b* *i* *l* *i* *r* *u* *b* *i* *n* > 34 * μ*mol/L with ALT or *A* *S* *T* > 2 times the upper limit of normal, thereby emphasizing concomitant hepatocellular injury. The third employs a higher bilirubin threshold (> 68–70 *μ*mol/L or > 4.0 mg/dL), with or without transaminase elevation.

## 2. Limitations of Conventional Biomarkers

### 2.1. ALT and AST

ALT and AST are widely utilized biochemical tests for assessing liver function, having been in use since the 1950s and remaining standard practice today. Numerous studies have demonstrated that patients with SALI exhibit markedly higher ALT levels than those only suffering from sepsis. However, there are several limitations associated with ALT and AST testing. Firstly, these enzymes lack specificity [4]. While primarily associated with liver function, ALT is also present in skeletal muscle and cardiac tissue, leading to potential elevations during myocardial infarction or other muscular injuries [5]. Similarly, AST is predominantly found in the myocardium and serves as a crucial marker for myocardial infarction and myocarditis. Secondly, although an elevated ALT level is one of the key criteria for diagnosing SALI, it does not facilitate early identification of this condition [6]. Thirdly, both ALT and AST possess relatively long half‐lives. It is particularly notable that the half‐life of ALT can extend to 47 h, which makes them less effective in the real‐time monitoring of the progression of liver injury [7].

### 2.2. Bilirubin

Bilirubin is one of the most frequently utilized biomarkers for assessing liver function and is employed in the SOFA. The Save Sepsis Campaign (SSC) guidelines suggest that serum bilirubin *c* *o* *n* *c* *e* *n* *t* *r* *a* *t* *i* *o* *n* *s* > 2 mg/dL (> 34.2 *μ*mol/L) be utilized as an indicator for identifying liver dysfunction. Another article defines SALI as a serum total bilirubin exceeding 4 mg/dL (> 70 *μ*mol/L). Although there is currently no agreed‐upon diagnostic criteria for SALI, most studies have tended to incorporate serum bilirubin levels in their criteria. Nevertheless, bilirubin still has the issue of a lack of specificity; for instance, hemolysis, drugs, and so forth, can result in the elevation of bilirubin. Furthermore, bilirubin cannot distinguish acute liver dysfunction caused by sepsis from preexisting chronic liver disease [8]. These are also reasons to attempt to discover more novel biomarkers.

Although ALT, AST, and bilirubin are the only biomarkers universally available around the clock in nearly all intensive care units, with negligible cost and turnaround times of less than 1 h, their lack of specificity and delayed kinetics remain major drawbacks. These limitations continue to fuel the search for more accurate markers. Ultimately, any novel biomarker must demonstrate clear and substantial clinical benefit over these inexpensive, immediately accessible conventional tests. Only then can its routine use in the ICU be justified.

## 3. Emerging Hepatocyte‐Specific Injury Biomarkers

### 3.1. miR‐122

MicroRNAs are single‐stranded noncoding RNAs that regulate gene expression by degrading mRNAs or inhibiting their translation. Owing to their encapsulation in extracellular vesicles or association with proteins, circulating miRNAs exhibit remarkable stability in blood, making them highly attractive as biomarkers [9]. Hundreds of miRNAs have been implicated in sepsis‐related organ dysfunction, with miR‐122, miR‐155, miR‐30a, and miR‐103a‐3p showing particularly strong associations with SALI [10].

Among these, miR‐122 is by far the most abundant liver‐specific miRNA, accounting for approximately 70% of the total hepatic miRNA pool [11]. It is rapidly released into the circulation upon hepatocyte injury and has already been validated as a sensitive and specific diagnostic marker for drug‐induced liver injury (DILI). Importantly, serum miR‐122 demonstrates high liver specificity and remains unaffected by renal impairment [12]. In the context of sepsis, miR‐122 performs exceptionally well. In a cohort of 108 septic patients and 20 nonseptic controls, miR‐122 not only discriminated infection from sepsis but also emerged as an early independent predictor of 30‐day mortality. Compared with nonseptic controls, miR‐122 levels rose sixfold in survivors and 40‐fold in nonsurvivors [13]. Another study comparing patients with various liver diseases, severe sepsis, and healthy controls found that miR‐122 was upregulated across all hepatic conditions; however, the most pronounced elevation occurred in patients with acute liver dysfunction secondary to sepsis [14]. Collectively, these findings establish miR‐122 as one of the most promising circulating biomarkers for the early detection and prognostic stratification of SALI.

Overall, miR‐122 is currently one of the most liver‐specific and the earliest rising biomarkers in the SALI cycle, with performance far exceeding that of ALT/AST in multiple studies. However, its translation into clinical practice faces significant limitations. The detection process requires RNA extraction, reverse transcription, and qRT‐PCR or ddPCR, leading to long turnaround times, high per‐test costs, and a reliance on specialized molecular laboratories—resources rarely available on‐site in most ICUs. Furthermore, the lack of standardized reference materials or commercial kits confines its current use primarily to research settings. Until a fully automated, rapid, and cost‐effective detection platform is developed, miR‐122 will remain a powerful research tool rather than a routine clinical test.

### 3.2. Hyaluronic Acid (HA)

HA is a key component of the extracellular matrix and is particularly abundant in the liver [15]. It is synthesized primarily by hepatic stellate cells and cleared almost exclusively by sinusoidal endothelial cells. For over four decades, serum HA has been recognized as a sensitive marker of liver fibrogenesis and endothelial dysfunction. In chronic liver diseases such as viral hepatitis, alcoholic liver disease, and nonalcoholic fatty liver disease, HA is one of the best‐validated noninvasive serum markers of fibrosis, offering higher sensitivity and specificity than many conventional parameters while avoiding the risks of liver biopsy [16, 17].

In the setting of critical illness, HA also reflects acute sinusoidal endothelial and global synthetic dysfunction. In a large cohort of 1096 critically ill patients, Jensen and colleagues compared HA with bilirubin, albumin, and the international normalized ratio (INR). HA exhibited the greatest dynamic range and most accurately captured the full spectrum of liver impairment. Among HA, bilirubin, and the Model for End‐Stage Liver Disease (MELD) score, HA showed the strongest and most linear association with mortality risk [18]. These findings suggest that HA is valuable not only for the early detection of SALI but also for tracking disease progression and predicting outcome.

Nevertheless, HA has limitations in the specific context of sepsis. Elevated levels may partly originate from bacterial wall‐derived HA fragments rather than solely from impaired hepatic clearance [9, 19]. This extra‐hepatic contribution reduces diagnostic specificity for SALI and underscores the need for further validation studies before HA can be recommended as a standalone biomarker in septic patients.

### 3.3. Glutamate Dehydrogenase (GLDH)

GLDH is a mitochondrial enzyme involved in amino acid metabolism and is most abundantly expressed in hepatocytes [20]. Although present in other tissues such as the brain and kidney, its serum levels are overwhelmingly liver‐specific and are not influenced by skeletal muscle or cardiac injury, unlike ALT and AST. In a comprehensive evaluation of candidate markers for DILI, GLDH and miR‐122 outperformed all others. Notably, GLDH proved superior to miR‐122 in correctly identifying true DILI cases and demonstrated faster normalization during recovery from acetaminophen (APAP) overdose, even when ALT remained elevated [21]. A subsequent study employing random forest modeling confirmed that GLDH, together with keratin‐18 (K18) and miR‐122, provided the highest diagnostic value among tested biomarkers. A three‐marker model (GLDH + K18 + miR‐122) accurately distinguished hepatocellular injury from muscle, pancreatic, or renal damage and clearly separated healthy individuals from those with liver injury—advantages that ALT and AST alone could not achieve [19].

These properties—high liver specificity, rapid kinetics, independence from extra‐hepatic sources, and proven performance in multimarker panels—position GLDH as an exceptionally strong candidate for early diagnosis and monitoring of SALI, either in combination with traditional markers to overcome their limitations or within novel composite panels to maximize sensitivity and specificity.

## 4. Mechanism‐Reflecting and Prognostic Biomarkers

### 4.1. Other MicroRNAs

Beyond miR‐122, several additional microRNAs have been linked to the pathogenesis and potential diagnosis of SALI.

miR‐155 is markedly upregulated during sepsis and contributes to oxidative stress and hepatocyte apoptosis. In LPS‐challenged mice, administration of a miR‐155 antagomir significantly improved survival and reduced ALT elevation by targeting Nrf2, thereby attenuating endoplasmic reticulum stress, mitochondrial dysfunction, and apoptosis [9]. Although miR‐155 has shown excellent diagnostic performance in hepatitis C‐related hepatocellular carcinoma (sensitivity 72.4% and specificity 95.2%) [22], its role as a circulating biomarker specifically for SALI remains preliminary and requires further clinical validation.

miR‐30a levels are significantly reduced in septic patients compared with healthy individuals or those with SIRS alone, suggesting diagnostic potential [23]. Experimental studies in septic rats demonstrated that upregulation of miR‐30a, achieved through SOCS‐1 inhibition, suppressed hepatocyte proliferation, promoted apoptosis, and impaired cell cycle progression, confirming its active participation in SALI progression [24].

miR‐103a‐3p exerts protective effects by directly targeting HMGB1. In murine sepsis models, administration of miR‐103a‐3p agomir preserved mitochondrial integrity, reduced hepatocyte apoptosis, and improved liver histology [25, 26]. Importantly, the antiapoptotic benefit was completely reversed by subsequent HMGB1 overexpression, verifying the mechanistic axis [25].

Collectively, miR‐155, miR‐30a, and miR‐103a‐3p highlight the regulatory complexity of microRNAs in SALI. While their therapeutic modulation appears promising in preclinical models, current evidence for their standalone diagnostic or prognostic utility in human sepsis is limited compared with miR‐122, and larger clinical studies are needed. Furthermore, all three miRNAs face the same practical barriers as miR‐122: complex preanalytical requirements, long turnaround times, high cost, and complete absence of clinical‐grade assays. Their current role is therefore restricted to mechanistic studies and therapeutic target validation rather than clinical decision‐making.

### 4.2. Differentially Expressed Genes (DEGs)

The identification of disease‐specific biomarkers through DEGs has become a widely adopted strategy in both oncology and critical illness. This approach has also been successfully extended to sepsis and various liver disorders. For instance, Li et al. revealed that MAPK14, identified through ferroptosis‐related DEGs, significantly influences the inflammatory response in pediatric sepsis and holds diagnostic value [27]. Similarly, Ke et al. confirmed that YOD1, GADD45A, BCL11B, and IL1R2 serve as robust prognostic biomarkers in adult sepsis cohorts [28]. In chronic liver disease, Govaere et al. identified AKR1B10 and GDF15 as progression markers in nonalcoholic fatty liver disease using DEG analysis [29].

In the specific context of SALI, Zhou et al. conducted a focused bioinformatics screening of 21 candidate DEGs derived from septic liver tissue. Strikingly, only TNFRSF1A demonstrated liver‐specific differential expression, strongly supporting its potential as a highly selective biomarker for SALI [30]. TNFRSF1A, the type I receptor for TNF‐*α*, is ubiquitously expressed and mediates critical immune functions through formation of distinct signaling complexes that trigger inflammation, immune defense, and apoptosis [31]. Excessive TNF‐*α* signaling is a well‐established driver of hepatocyte injury, and inhibition of this pathway effectively attenuates liver damage in multiple models [32].

In septic mice, TNFRSF1A protein was detectable in liver, lung, and kidney tissues; however, only hepatic expression showed a significant decrease, reinforcing organ‐specific dysregulation in SALI [30]. Mechanistically, TNFRSF1A promotes inflammatory injury and apoptosis via multiple downstream routes, including the mTOR signaling pathway [33]. Interestingly, regulated shedding of TNFRSF1A limits overwhelming inflammation through the MyD88–iNOS–cGMP axis. In a hepatocyte‐specific MyD88‐knockout sepsis model, impaired TNFRSF1A shedding exacerbated liver injury, highlighting its protective role when appropriately controlled [34].

Thus, among numerous DEGs evaluated in sepsis and liver injury, TNFRSF1A stands out for its liver‐restricted expression pattern and central involvement in key pathogenic processes of SALI. These findings position TNFRSF1A as one of the most promising DEG‐derived candidates for future diagnostic, prognostic, and even therapeutic applications in SALI.

### 4.3. HMGB1

HMGB1 is a highly conserved nuclear protein expressed in nearly all cell types [35]. Under physiological conditions, it stabilizes nucleosomes and facilitates transcription. However, during cellular stress or death, HMGB1 is actively secreted by immune cells or passively released from necrotic hepatocytes, acting as a prototypic DAMP that sustains inflammation via RAGE and TLR4 signaling [36]. Extensive evidence confirms its pivotal role in both acute and chronic liver diseases, where massive passive release from injured hepatocytes drives persistent inflammation and exacerbates tissue damage [37].

Pyroptosis is now recognized as a central mechanism in SALI [38]. The canonical pathway involves NLRP3 inflammasome activation, ASC recruitment, pro‐caspase‐1 cleavage, and subsequent gasdermin D (GSDMD) processing, resulting in membrane pore formation and lytic cell death [39]. Caspase‐1 expression and activity increase markedly during sepsis [40], and genetic knockout of HMGB1 dramatically reduces NLRP3/caspase‐1 levels, underscoring its upstream regulatory role [41]. Furthermore, miR‐103a‐3p ameliorates SALI in septic rats by directly targeting HMGB1, reinforcing its mechanistic importance and therapeutic potential [25]. Clinical and experimental studies consistently demonstrate elevated HMGB1 in SALI patients and septic mice, with levels correlating closely with the severity of liver injury [37].

HMGB1 has been extensively investigated as a biomarker across diverse liver pathologies, including fibrosis, hepatocellular carcinoma, ischaemia–reperfusion injury, and DILI [42]. A meta‐analysis of 16 studies confirmed significantly higher serum HMGB1 in severe hepatitis B and acute‐on‐chronic liver failure, supporting its diagnostic utility [43]. In APAP‐induced liver injury, HMGB1 accurately reflects hepatocyte necrosis [44]. However, a major limitation is its relatively late rise; significant elevations typically occur only in severe or advanced injury, restricting its value for early detection of SALI [44]. Despite this drawback, HMGB1 remains a robust marker of inflammatory severity and a promising therapeutic target in SALI.

### 4.4. PPAR‐*α*

PPAR‐*α* is a ligand‐activated transcription factor of the nuclear receptor superfamily that is highly expressed in tissues with active fatty acid catabolism, including the liver, heart, and kidney [45]. In hepatocytes, PPAR‐*α* orchestrates all three major pathways of fatty acid oxidation (peroxisomal *β*‐oxidation, mitochondrial *β*‐oxidation, and microsomal *ω*‐oxidation), thereby maintaining lipid and energy homeostasis. It also modulates lipoprotein metabolism, inflammatory responses, and insulin sensitivity [46]. PPAR‐*α* is capable of alleviating acute liver injury. Some researchers have utilized fenofibrate (a PPAR‐*α* agonist) to inhibit the acute‐phase response in the livers of mice [47]. Furthermore, studies have indicated that PPAR‐*α* also plays a certain regulatory role in hepatic glucose metabolism. In mice treated with fenofibrate, the flux of glucokinase decreased, suggesting that the activation of PPAR‐*α* has a negative influence on hepatic glucose uptake and glycolysis [48]. Consequently, the role of PPAR‐*α* in diseases such as nonalcoholic steatohepatitis and cirrhosis has received extensive attention.

Emerging evidence highlights a critical protective role for PPAR‐*α* in SALI. In cecal ligation and puncture (CLP) models, sepsis rapidly suppresses hepatic PPAR‐*α* mRNA and protein levels, leading to accumulation of free fatty acids, impaired *β*‐oxidation, and hepatocyte toxicity [49]. Importantly, pharmacological activation of residual PPAR‐*α* after sepsis onset significantly improves survival [49]. Subsequent studies using gypenoside XLIX, a natural PPAR‐*α* agonist, confirmed that PPAR‐*α* activation inhibits inflammatory responses and acute‐phase reactions in CLP mice, reduces hepatic lipid accumulation, and attenuates liver damage via the NF‐*κ*B/PPAR‐*α*/NLRP3 axis [50]. Recent work has further linked PPAR‐*α* to suppression of ferroptosis in sepsis by upregulating glutathione peroxidase 4 (GPX4) and downregulating transferrin receptor expression.

Although circulating PPAR‐*α* protein or its direct downstream metabolites are not yet established as routine biomarkers, the consistent downregulation of hepatic PPAR‐*α* expression and the clear survival benefit conferred by its pharmacological activation suggest that PPAR‐*α* pathway activity may serve as both a prognostic indicator and a therapeutic target in SALI. Further clinical studies are needed to translate these preclinical findings into measurable biomarkers or targeted interventions.

### 4.5. Macrophage Colony‐Stimulating Factor (CSF1)

Macrophages constitute a cornerstone of innate immunity, playing essential roles in pathogen clearance, immune regulation, and tissue homeostasis [51]. In the liver, the macrophage pool comprises resident Kupffer cells (KCs) and monocyte‐derived macrophages (MDMs) recruited during injury [52]. These cells exert context‐dependent effects in both acute and chronic liver diseases: They initially eliminate pathogens and debris but can subsequently amplify inflammation or promote resolution and repair [53–55].

In SALI, resident KCs are rapidly activated and often undergo necroptosis or depletion, whereas circulating monocytes are massively recruited and differentiate into proinflammatory MDMs [56]. This dynamic shift is tightly regulated by CSF1 (also known as M‐CSF), which is markedly elevated in the circulation during liver injury due to impaired hepatic clearance and increased production by multiple cell types [57]. CSF1 acts via the macrophage colony‐stimulating factor receptor (CSF1R) to drive survival, proliferation, and differentiation of both resident and MDM populations [58].

Elevated circulating CSF1 has already been linked to the severity of various liver insults. Its prognostic value has been demonstrated in chronic hepatitis B, DILI, and acute liver failure [59]. Notably, Stutchfield et al. observed that serum CSF1 rises proportionally to the extent of liver resection, whereas persistently low CSF1 in patients with acute liver failure predicts higher mortality [60]. These findings suggest that CSF1 concentration reflects the intensity of macrophage activation and the balance between injurious and reparative responses.

In conclusion, although direct evidence in human SALI remains limited, the central role of dysregulated macrophage responses in sepsis pathophysiology, combined with the consistent association between circulating CSF1 and outcome in other acute liver injuries, strongly supports its potential as a biomarker for diagnosing, monitoring, and prognosticating SALI. Prospective clinical studies measuring serial CSF1 levels in septic patients with liver dysfunction are now warranted to establish its diagnostic and predictive performance.

## 5. Multimarker Approaches and Future Directions

At present, no single biomarker can fully meet the needs of early diagnosis, severity assessment, and prognostic evaluation of SALI. Traditional markers such as ALT, AST, and bilirubin suffer from poor specificity and delayed elevation, while even the most promising new candidates (miR‐122, GLDH, K18, HA, HMGB1, and TNFRSF1A) have their own limitations, including differences in release timing, potential interference from extra‐hepatic sources, or inconsistent performance across patient populations.

Strong evidence now shows that combining several complementary biomarkers significantly improves both diagnostic and prognostic accuracy. The most robust and reproducible example is the panel of GLDH + miR‐122 + K18 (total K18 or caspase‐cleaved K18), which has repeatedly achieved AUC values above 0.93 in studies of drug‐induced and APAP‐induced liver injury and reliably distinguishes genuine hepatocellular injury from muscle, pancreatic, or renal damage. Although these data originate mainly from nonseptic cohorts, the underlying mechanisms of hepatocyte injury are highly similar to those in SALI, making the findings directly relevant.

Recent preliminary studies in sepsis patients further confirm that combining rapidly released liver‐specific markers (miR‐122 and GLDH) with later‐appearing inflammation‐related markers (HMGB1, TNFRSF1A, and CSF1) simultaneously improves early detection rates and the ability to predict progression to severe liver dysfunction. When these biomarkers are integrated with routine clinical parameters (e.g., SOFA score, lactate, and platelet count) using straightforward machine learning models, predictive performance consistently surpasses that of existing scoring systems.

In conclusion, relying solely on ALT and bilirubin is no longer sufficient. The rational combination of fast, highly liver‐specific injury markers with selected inflammation‐ and mechanism‐related markers currently represents the most practical and effective way to substantially improve early diagnosis and prognostic accuracy in SALI. The highest priority now is rigorous clinical validation of these panels in real‐world sepsis cohorts so that they can be rapidly translated into routine critical care practice.

## 6. Conclusions

SALI remains a frequent and ominous complication in critically ill patients, yet it is still diagnosed late and imprecisely with conventional liver tests. This review highlights that several new biomarkers—miR‐122, GLDH, K18, HA, HMGB1, soluble TNFRSF1A, and CSF1—each address specific shortcomings of ALT, AST, and bilirubin, offering greater liver specificity, faster kinetics, or deeper insight into underlying inflammatory and cell death mechanisms. Although none of these markers is perfect in isolation, their complementary strengths provide a clear opportunity to move beyond single‐analyte testing. The available evidence strongly supports the superiority of rationally combined panels over any individual marker or traditional liver function test for the early recognition and risk stratification of SALI.

**Figure 1:**
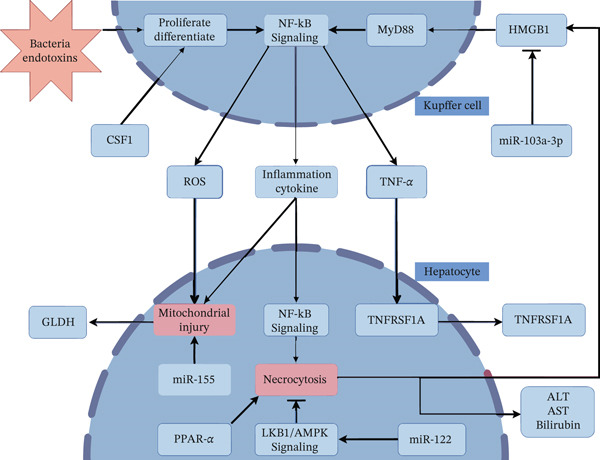
Biomarkers of inflammatory damage mechanisms in SALI.

As summarized in Table 1 and illustrated in Figure 1, each biomarker discussed in this review has distinct advantages and limitations in the context of SALI. These findings underscore the importance of a multimarker approach that combines complementary biomarkers to improve diagnostic accuracy and prognostic prediction. Large‐scale clinical validation of these multimarker approaches is now the critical next step. Successful implementation will equip intensivists with objective, timely, and actionable information, transforming our ability to detect, monitor, and manage SALI in routine critical care practice.

**Table 1:** Biomarkers in SAL.

Biomarker	Relevant mechanism	Advantages	Limitations
miR‐122	Inhibits LKB1/AMPK signaling	Highly specific for liver injuryEarly independent predictor of 30‐day mortalityGood stability in blood	Cannot differentiate SALI from other acute liver injuries
miR‐155	Promotes ER stress, mitochondrial dysfunction, and apoptosis via oxidative stress	Good stability in bloodDiagnostic potential in hepatitis C‐related HCC	Limited clinical studies in SALI
miR‐103a‐3p	Inhibits HMGBI function	Good stability in bloodProtective effects in sepsis models	Limited clinical studies in SALI
HMGB1	Activates NF‐*κ*B/NLRP3 signaling	Dual value as biomarker and therapeutic targetReflects inflammatory severity	Elevates late in severe injury, limiting early diagnosis
PPAR‐*α*	Activates NF‐*κ*B/NLRP3 signaling	Dual value as biomarker and therapeutic targetCritical protective role I sepsis	Not liver specific (also in the heart and kidney)
CSF1	Activates NF‐*κ*B signaling	Reflects degree of inflammationValue in assessing extent of liver damage	Limited specificity for liver injury
TNFRSF1A	MyD88 promotes its shedding into bloodstream	Highly specific expression in SALICentral to pathogenic processes	Complex mechanisms require further investigation

## Author Contributions

Yao‐zheng Cai conceived the idea and designed the manuscript. Yao‐zheng Cai, Jiang‐tao Chen, Le‐xin Fang, and Yue‐ping Ding contributed to interpreting data, drafting the manuscript, and revising and editing the manuscript for intellectual content.

## Funding

No funding was received for this manuscript.

## Disclosure

All authors gave final approval for the submission.

## Conflicts of Interest

The authors declare no conflicts of interest.

## Data Availability

Data sharing is not applicable to this article as no datasets were generated or analyzed during the current study.
